# Adipose tissue dysfunction serum markers are associated with high density lipoprotein size and glycation in the early stages of type 2 diabetes

**DOI:** 10.1186/s12944-023-01847-7

**Published:** 2023-07-01

**Authors:** Esteban Jorge-Galarza, Aida Medina-Urrutia, Juan Reyes-Barrera, Margarita Torres-Tamayo, Luis Felipe Montaño-Estrada, Araceli Páez-Arenas, Felipe Massó-Rojas, Juan Gabriel Juárez-Rojas

**Affiliations:** 1 Departamento de Endocrinología, Instituto Nacional de Cardiología Ignacio Chávez, Mexico City, Mexico; 2 Laboratorio de Inmunobiología, Departamento de Biología Celular y Tisular, Facultad de Medicina, Universidad Nacional Autónoma de México (UNAM), Mexico City, Mexico; 3 Posgrado en Ciencias Biológicas, Unidad de Posgrado, Universidad Nacional Autónoma de México (UNAM), Mexico City, Mexico; 4 Laboratorio de Medicina Traslacional, Instituto Nacional de Cardiología Ignacio Chávez, Mexico City, Mexico

**Keywords:** Type 2 diabetes, High-density lipoproteins, Adipose tissue dysfunction, Advanced glycation end products, And high-density lipoprotein size

## Abstract

**Background:**

High-density lipoproteins (HDLs) have antiatherogenic properties related to their chemical structure. Adipose tissue (AT) influences HDL reverse cholesterol transport and plasma HDL cholesterol levels. However, whether AT dysfunction affects HDL subpopulations and their glycation in early type 2 diabetes (T2D) is still unknown.

**Objective:**

To investigate the association of inflammation and AT dysfunction serum markers with the size and glycation of HDLs in normoglycemic, prediabetes, and T2D subjects.

**Methods:**

We assessed HDL particle size and advanced glycation end-product (AGE) content in HDLs isolated from normoglycemic (n = 17), prediabetes (n = 17), and recently T2D-diagnosed (n = 18) subjects. Insulin, adiponectin, and plasminogen activator inhibitor 1 (PAI-1) were determined using the Bio-Rad Multiplex Platform, and free fatty acids (FFAs) and high sensitivity C-reactive protein (hs-CRP) were determined by standard procedures. The AT insulin resistance (ATIR) index and ATIR/adiponectin and adiponectin/leptin ratios were calculated.

**Results:**

HDL was progressively smaller (nm) and enriched with AGE (mg-BSA-AGE/mg protein) according to the glucose categories: 8.49 and 7.5 in normoglycemic subjects, 8.44 and 12.4 in prediabetic subjects, and 8.32 and 14.3 in T2D subjects (*P* = 0.033 and *P* = 0.009 for size and AGE, respectively). In multivariable regression analysis, the ATIR/adiponectin ratio was inversely associated with HDL size (β = -0.257, *P* = 0.046), and the ATIR ratio was directly associated with HDL glycation (β = 0.387, *P* = 0.036). In contrast, adiponectin and the adiponectin/leptin ratio were not associated with alterations in HDL particles. Furthermore, HDL size was associated with resistin (β = -0.348, *P* = 0.007) and PAI-1 (β = -0.324, *P* = 0.004). HDL and AGE were related to insulin concentrations (β = 0.458, *P* = 0.015). Analyses were adjusted for age, sex, body mass index, triglycerides, and HDL-cholesterol.

**Conclusion:**

HDL size was significantly associated with the ATIR/adiponectin ratio and inflammation, whereas glycation was more strongly related to the ATIR index. These findings have important implications for the management and prevention of cardiovascular disease in T2D patients.

## Background

Beyond its role as an energy reservoir, white adipose tissue (AT) is a metabolically active endocrine organ that regulates blood lipid levels, blood pressure, inflammation, and angiogenesis [1]. This tissue can expand by increasing the cell number (hyperplasia) and/or size (hypertrophy) of mature adipocytes [2]. In a chronic condition of excessive energy intake, adipocytes can reach a critical cell size. A larger average size of adipocytes accompanied by lower oxygen tension (hypoxia) is usually observed in the adipose tissue of obese subjects compared to lean subjects [3, 4].

The convergence of hypoxia, insulin resistance, and low-grade chronic inflammation contributes to AT dysfunction, characterized by a shift in the adipocyte secretory profile toward a proinflammatory phenotype. Hypertrophic adipocytes can produce cytokines such as tumor necrosis factor alpha (TNF-alpha), interleukins 6 (IL-6) and 8 (IL-8), and monocyte chemoattractant protein 1 (MCP-1) [5], which have proinflammatory action and induce immune cells such as macrophages and T cells. In this microenvironment, adipocytes reduce adiponectin and increase leptin and resistin secretion [6]. These cellular stress responses can drive adipocytes into apoptosis if the conditions persist [7].

Adiponectin is a pleiotropic hormone derived from adipocytes and has anti-inflammatory, antidiabetic, and antiatherogenic properties [8]. The serum concentration of this adipocytokine is decreased in the metabolically unhealthy obese phenotype compared to the metabolically obese healthy phenotype or normal-weight individuals [9]. Therefore, it is considered a good marker of AT quality [10]. On the other hand, leptin regulates physiological energy processes through satiety, increasing energy expenditure and promoting adipose tissue lipolysis. Although leptin concentrations increase with increasing fat volume, elevated levels of this hormone are associated with insulin resistance [11], inflammation, and AT lipolysis, probably through resistance to its catabolic effects [2, 12]. Based on the above information, the adiponectin/leptin ratio has been proposed as a useful clinical marker to identify adipose tissue dysfunction [8, 13]. This index has been shown to be a strong marker of insulin resistance, even better than the homeostatic model assessment of insulin resistance (HOMA-IR), quantitative insulin sensitivity check index, or fasting insulin plasma levels [14]. In addition, the index has been related to abnormal concentrations of high-density lipoprotein cholesterol (HDL-C) and triglycerides. The latter effect was independent of body mass index (BMI), and its association was stronger than with adiponectin or leptin serum levels [15]. The AT insulin resistance index (ATIR) is another marker of adipose tissue quality that reflects the metabolic dysregulation of lipolysis and can be easily estimated by the serum levels of insulin multiplied by free fatty acids (FFAs) [16, 17]. This ratio has been associated with nonalcoholic fatty liver disease [16], insulin resistance, impaired fasting glucose, and early type 2 diabetes mellitus (T2D) [18, 19].

High-density lipoproteins (HDLs) are heterogeneous complexes varying in shape, size, and lipid and protein content. Because of their antioxidant, anti-inflammatory, antiapoptotic, antithrombotic, and vasodilatory properties, these lipoproteins are considered atheroprotective. Furthermore, they can also promote cholesterol efflux from the macrophages of the subendothelial region, which prevents atherosclerosis development [20]. Previously, it has been reported that HDL subfractions leading to a smaller size are related to cardiovascular risk conditions, including T2D [21]. Moreover, triglyceride enrichment, cholesterol ester depletion, and HDL glycation are changes commonly found in T2D patients [22]. There is mounting evidence showing an interrelationship between adipose tissue and HDL metabolism, as adipocytes can play an active role in regulating total cholesterol balance and HDL production [23]. We previously reported that subjects with adipose tissue dysfunction, as determined by the combination of low adiponectin and elevated ATIR values, had smaller estimated HDL sizes [24]. The purpose of this study was to assess the association of inflammation and AT dysfunction serum markers with HDL size and the glycation of these particles in normoglycemic, prediabetic, and T2D subjects.

## Materials and methods

The studied population included a convenience sample of subjects from the control arm of the Genetics of Atherosclerosis Disease Study [24] and employees of the *Instituto Nacional de Cardiología Ignacio Chávez*, considering the following inclusion criteria: men and women aged 40 to 60 years, BMI ≤ 33 kg/m^2^, a previous fasting plasma glucose measurement, and those who did not self-report a personal history of cardiovascular, kidney, liver, or thyroid disease. Subjects under hypolipidemic and antihypertensive treatment were excluded. Elimination criteria included liver enzyme levels three times above the reference values for our population (aspartate aminotransferase (AST) > 126 IU/L and alanine aminotransferase (ALT) > 123 IU/L), triglycerides > 6.78 mmol/L (which can interfere with LDL-C calculation) and high-sensitivity C-reactive protein (hs-CRP) values > 10 mg/L to discard the presence of a possible acute inflammatory process. The final sample was stratified according to the American Diabetes Association [25] as follows: (1) normoglycemic (n = 18), with fasting glucose values < 5.6 mmol/L and plasma glucose < 7.8 mmol/L measured after 2 h of 75 g of oral glucose intake (oral glucose tolerance test [OGTT]); (2) prediabetes (n = 17), with fasting glucose values 5.6–6.9 mmol/L or glucose 7.8–11 mmol/L after OGTT; and (3) T2D (n = 18) included newly diagnosed patients with less than four years of evolution and those with fasting glucose values > 6.9 mmol/l or glucose > 11 mmol/L after OGTT. Seven T2D subjects were under metformin treatment, and two of them also received DPP4 inhibitors. To minimize possible bias due to pharmacological treatment, T2D patients suspended medication 72 h before the study under physician surveillance and continued treatment after the study. The Ethics and Research Committee of the *Instituto Nacional de Cardiología Ignacio Chávez* approved the study.

After fasting for 12 h, 20 mL of blood was collected to obtain plasma and serum. Blood samples were separated and immediately frozen at -80 °C until analysis. Total cholesterol, HDL-C, fasting glucose, triglycerides, apolipoprotein A1 (ApoA1), apolipoprotein B-100 (ApoB-100), hemoglobin A1C (HbA1C), AST, ALT, creatinine, and hs-CRP were determined by standard procedures with an automated analyzer (Roche Diagnostics, Manheim, Germany). The interassay coefficient of variation was less than 6% in all determinations. Low-density lipoprotein cholesterol (LDL-C) was calculated according to Delong et al. [26]. The glomerular filtration rate (GFR) was estimated using the Chronic Kidney Disease Epidemiology Collaboration formula. Insulin, adiponectin, leptin, resistin, C-peptide, and plasminogen activator inhibitor-1 (PAI-1) were determined using the Bio-Plex Multiplex Immunoassay System (Bio-Rad Laboratories, Hercules, CA, US). Serum-free fatty acids (FFAs) were determined with an enzymatic colorimetry assay (Wako Diagnostics, Osaka, Japan) with an interassay coefficient < 3%. The following serum markers were considered indicators of adipose tissue functionality: ATIR, calculated with the following formula: ATIR = FFA (mmol/L) X Insulin concentrations (µIU/L) [16, 18]. ATIR/adiponectin index and adiponectin/leptin ratio.

### HDL characterization

HDLs were isolated from the plasma by sequential ultracentrifugation using a potassium bromide solution (0.5 g/L EDTA) at a 1.21 g/mL density [27]. Total HDLs were dialyzed against PBS (10 mM, pH 7.4) and loaded onto a 4–25% native polyacrylamide gel to estimate the average size of HDL and the subclasses HDL2b, HDL2a, HDL3a, HDL3b, and HDL3c. The gels were analyzed by densitometry to express the subclasses in a relative proportion to 100%. HDL glycation of early (fructosamine), intermediate (dicarbonyls), and advanced glycation end products (AGEs) was determined. Fructosamine was quantified using colorimetric reagents in an automated autoanalyzer (Roche Diagnostics, Manheim, Germany). Dicarbonyls were determined as previously reported [28]. Briefly, the isolated HDLs were incubated with 2–4 dinitrophenyl-phenylhydrazine in 2.5 N HCl for 60 min at 25 °C and precipitated with 20% trichloroacetic acid (*w/v*). The pellet was dissolved in 6 M HCl-guanidine and incubated for 10 min at 37 °C to quantify the absorbance at 370 nm using a Biotek Synergy H1 spectrophotometer (Biotek Instruments, Winooski, VT, USA). The AGE concentration in HDL was quantified using a commercial competitive ELISA kit (OxiSelect™ STA-817, Cell Biolabs, Inc. San Diego, CA, USA). This method identifies carboxymethyl lysine and pentosidine adducts.

### Statistical analyses

For the present study, a sample size estimation analysis using HDL size as the primary variable [29], with a power of 80% and a confidence interval of 95%, was performed. The analysis indicated the need for 16 subjects to detect significant differences among the study groups. Continuous variables were evaluated for normality and expressed as the mean ± standard deviation or median (interquartile range), as appropriate. Categorical variables were reported as the number of subjects and their prevalence. Differences in means were compared using analysis of variance (ANOVA) with Bonferroni correction for multiple comparisons. The Kruskal‒Wallis test was used to compare medians, and the chi-square test was used to analyze prevalence values. Correlations were evaluated by univariate linear regression, calculating the standardized beta coefficient. Model 1 was adjusted for age and sex; model 2 corresponded to model 1 plus BMI, HDL-C, and triglycerides. A *P* value of < 0.05 was considered statistically significant. All analyses were performed using Stata 12 IC software (StataCorp LLC, College Station, TX, USA).

## Results

The clinical and biochemical features of the participants are summarized in Table 1. There was no statistically significant difference between groups in age, sex proportion, prevalence of smoking, plasma concentration of total cholesterol, LDL-cholesterol, triglycerides, ApoA1, ApoB-100, AST, ALT, and glomerular filtration rate. BMI was slightly higher in the prediabetic group than in the normoglycemic group (28.6 ± 4 kg/m^2^ vs. 25.3 ± 3 kg/m^2^, *P* < 0.063) and almost identical to that in the diabetic group (27.6 ± 4.6 kg/m^2^). As expected, HbA1c values were significantly higher in the prediabetic (5.82%) and diabetes (6.6%) groups than in the normoglycemic (5.47%) group (*P* < 0.001).

**Table 1:** Clinical and metabolic characteristics of the studied groups

	Normoglycemic n = 17	Prediabetes n = 17	T2D n = 18	*P* value
Age (years)	48.5 ± 6.3	52 ± 6.7	49.4 ± 7.5	0.295
Sex (male/female)	7/10	6/11	8/10	0.461
BMI (kg/m2)	25.3 ± 3.2	28.6 ± 4.2	27.6 ± 4.6	0.063
Smoking (%)	3 (17.7)	3 (17.7)	2 (11.1)	0.824
Hemoglobin A1C (%)	5.47 (5.2–5.6)	5.82 (5.7–5.9) *	6.6 (6.5–7.2) *†	< 0.001
Fasting Glucose (mmol/L)	4.86 (4.7–5.3)	5.61 (5.3–5.8) *	6.94 (5.9–8.2) *†	< 0.001
Total cholesterol (mmol/L)	4.91 ± 1.03	5.43 ± 1.4	4.56 ± 1.1	0.113
LDL cholesterol (mmol/L)	3.05 (2.6–3.6)	3.54 (2.9-4.0)	2.66 (2.3–3.2)	0.075
HDL cholesterol (mmol/L)	1.28 ± 0.34	1.19 ± 0.31	1.01 ± 0.34	0.059
Triglycerides (mmol/L)	1.27 (0.89–1.62)	1.69 (1.23-2.0)	1.74 (1.4–2.07) *	0.075
ApoA1 (g/L)	1.44 ± 0.22	1.39 ± 0.21	1.33 ± 0.26	0.399
ApoB-100 (g/L)	1.08 ± 0.29	1.24 ± 0.35	1.1 ± 0.26	0.557
AST (IU/L)	20 (17–25)	21 (17–23)	22 (17–25)	0.951
ALT (IU/L)	22 (15–39)	20 (16–26)	25 (20–35)	0.404
GFR (mL*min/1.73m^2^)	100 (85–104)	99 (93–102)	103 (84–109)	0.944

Values are expressed as the mean ± standard deviation, median (interquartile range), or number of subjects (%). HDL-C: high-density lipoprotein cholesterol; LDL-C: low-density lipoprotein cholesterol; AST: aspartate aminotransferase; ALT: alanine aminotransferase. GFR: glomerular filtration rate. * *P* < 0.05 vs. normoglycemic, † *P* < 0.05 vs. prediabetes; *P* value for means was calculated using ANOVA with Bonferroni correction. The Kruskal‒Wallis test was used to compare medians, and the chi-square test was used to analyze prevalence values

Among the AT dysfunction serum markers, ATIR and ATIR/adiponectin were higher in the abnormal glucose groups than in the normoglycemic group, with a significant difference in the T2D group (Table 2). Adiponectin and the adiponectin/leptin ratio showed a trend toward lower values in the abnormal glucose groups. On the other hand, insulin, HOMA-IR, and the ratio HOMA-IR/adiponectin were significantly higher in the T2D group than in the control group. Other glucose metabolism markers and inflammation molecules were not significantly different among groups.

**Table 2:** Markers of adipose tissue dysfunction, glucose, and inflammation in the studied groups

	Normoglycemic n = 17	Prediabetes n = 17	T2D n = 18	*P* value
*AT dysfunction markers*				
ATIR	4.12 ± 2.5	5.96 ± 2.8	7.85 ± 4.6 *	0.009
ATIR/adiponectin	0.52 (0.12–0.7)	0.74 (0.3–1.22)	1.1 (0.55–1.9) *	0.047
Adiponectin/leptin	4.22 (1.4–13.3)	2.8 (1.4–6.1)	2.4 (1.31-5)	0.564
Adiponectin (µg/mL)	8.48 (5.8–26)	8.99 (4.3–18)	7.0 (4.5–17)	0.808
*Glucose and lipid metabolism markers*				
Insulin (µIU/mL)	5.97 (5.3–10.8)	9.97 (6.7–13) *	12 (8.1–18) *	0.027
HOMA-IR	1.28 (1.1–2.2)	2.5 (1.6–3.4) *	3.8 (2.8–6.2) *†	< 0.001
HOMA-IR/Adiponectin	0.15 (0.04–0.40)	0.32 (0.1–0.43)	0.70 (0.20–1.4) *	0.029
C-Peptide (pg/mL)	607 ± 259	663 ± 241	790 ± 415	0.391
Leptin (ng/mL)	2.58 (0.8–6.1)	4.6 (3.0-6.1)	3.1 (0.92–4.3)	0.265
FFA (mmol/L)	0.56 ± 0.18	0.59 ± 0.14	0.61 ± 0.2	0.544
*Inflammatory markers*				
Resistin (ng/mL)	3.8 ± 1.4	3.5 ± 0.7	3.6 ± 1.3	0.822
PAI-1 (ng/mL)	7.4 ± 2.6	7.1 ± 2.3	7.6 ± 2.1	0.809
hs-CRP (mg/L)	1.41 (0.8–2.2)	1.02 (0.7–1.6)	1.6 (0.92-5.0)	0.504

Values are expressed as the mean ± standard deviation, median (interquartile range)

* *P* < 0.05 vs. normoglycemic, † *P* < 0.05 vs. prediabetes. ATIR: adipose tissue insulin resistance. HOMA-IR; homeostasis model of assessment-insulin resistance; FFA: free fatty acids. PAI-1; plasminogen activator inhibitor-1. hsCRP: high sensitivity C-reactive protein; *P* value for means was calculated using ANOVA with Bonferroni correction. The Kruskal‒Wallis test was used to compare medians

HDL size was significantly smaller in T2D subjects than in the prediabetic and normoglycemic individuals (8.32 nm vs. 8.44 and 8.49 nm, respectively, *P* = 0.033) due to a lower proportion of HDL2a and an increase in HDL3c in the T2D individuals (Table 3). In addition, HDLs had a higher glycation tendency, especially in AGE content, with values of 7.5, 12.4, and 14.3 (mg-BSA-AGE/mg protein) in normoglycemic, prediabetic, and T2D individuals, respectively (*P* = 0.009).

**Table 3:** HDL subpopulations and glycation according to the studied groups

	Normoglycemic n = 17	Prediabetes n = 17	T2D n = 18	*P* value
*HDL subpopulations*				
HDL2b (%)	8.5 ± 3.6	7.7 ± 2.7	6.2 ± 2.6	0.074
HDL2a (%)	17.2 ± 3.7	15.2 ± 4.3	13.4 ± 5.1 *	0.047
HDL3a (%)	23.2 ± 2.3	24.0 ± 2.4 *	21.2 ± 4.4 †	0.033
HDL3b (%)	25.4 ± 2.6	27.0 ± 2.5	26.4 ± 2.7	0.224
HDL3c (%)	25.8 ± 6.9	26.9 ± 5.8	32.8 ± 11 *	0.028
HDL mean diameter (nm)	8.49 ± 0.20	8.44 ± 0.17	8.32 ± 0.20 *	0.033
*HDL glycation*				
HDL- fructosamine	4.55 ± 0.79	4.67 ± 0.85	5.18 ± 1.1	0.139
HDL- carbonyls	96.4 ± 22	98.5 ± 29	102.3 ± 24	0.824
HDL-AGE	7.5 (6.8–9.9)	12.4 (8.2–16.3)	14.3 (8.8–19) *†	0.009

Values are expressed as the mean ± standard deviation, median (interquartile range)

* P < 0.05 vs. normoglycemic, † p < 0.05 vs. prediabetes. The *P* value for means was calculated by ANOVA with Bonferroni correction, and that for medians was calculated by the Kruskal‒Wallis test. HDL: high density lipoproteins; AGE: advanced glycation products

**Fig. 1:**
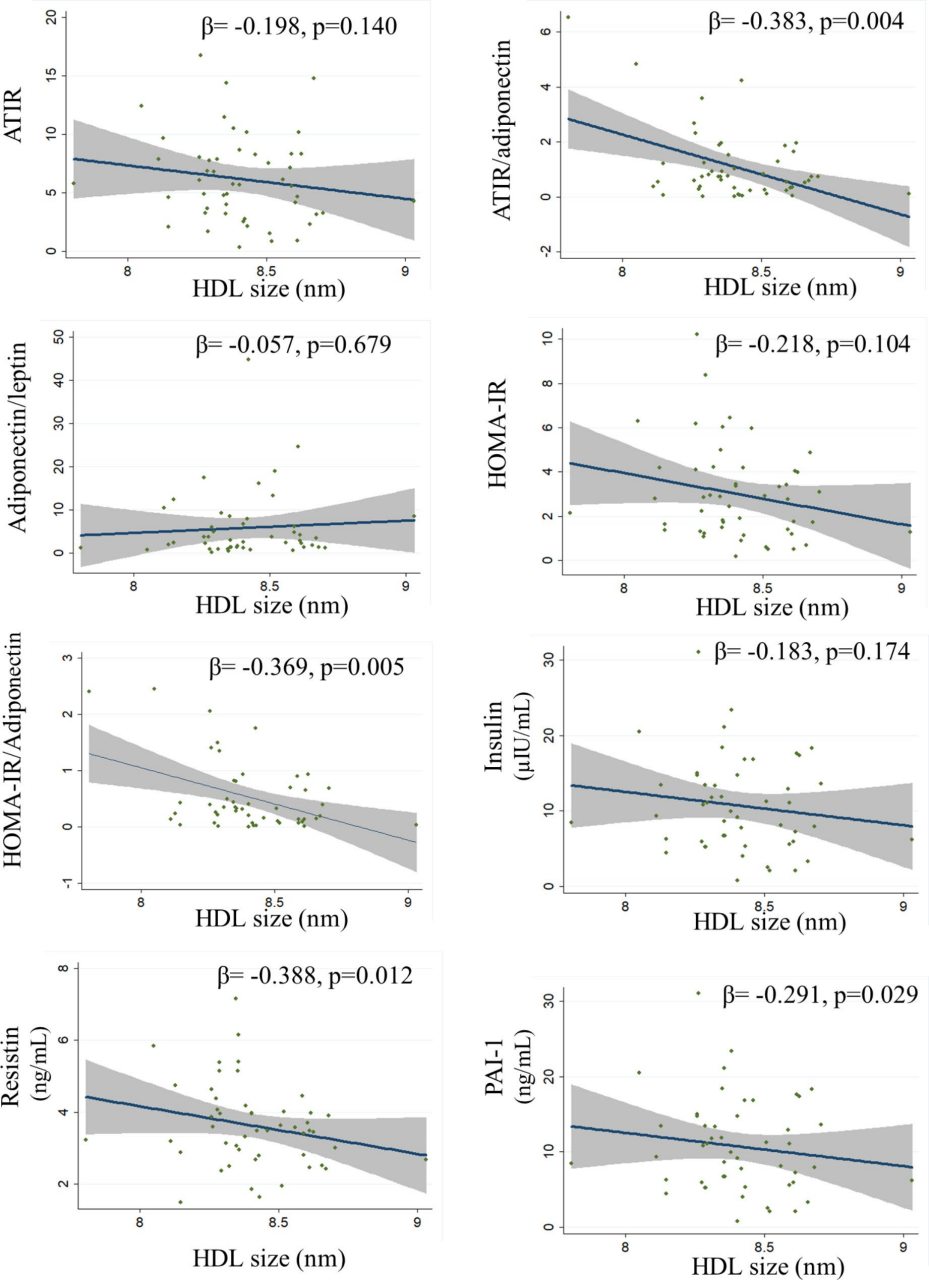
Correlations of HDL size with adipose tissue dysfunction serum markers and inflammation. Analyses were made for the whole population. Values are expressed as standardized beta coefficients in univariable regression. ATIR: adipose tissue insulin resistance. HOMA-IR; homeostasis model of assessment-insulin resistance; PAI-1; plasminogen activator inhibitor-1

**Fig. 2:**
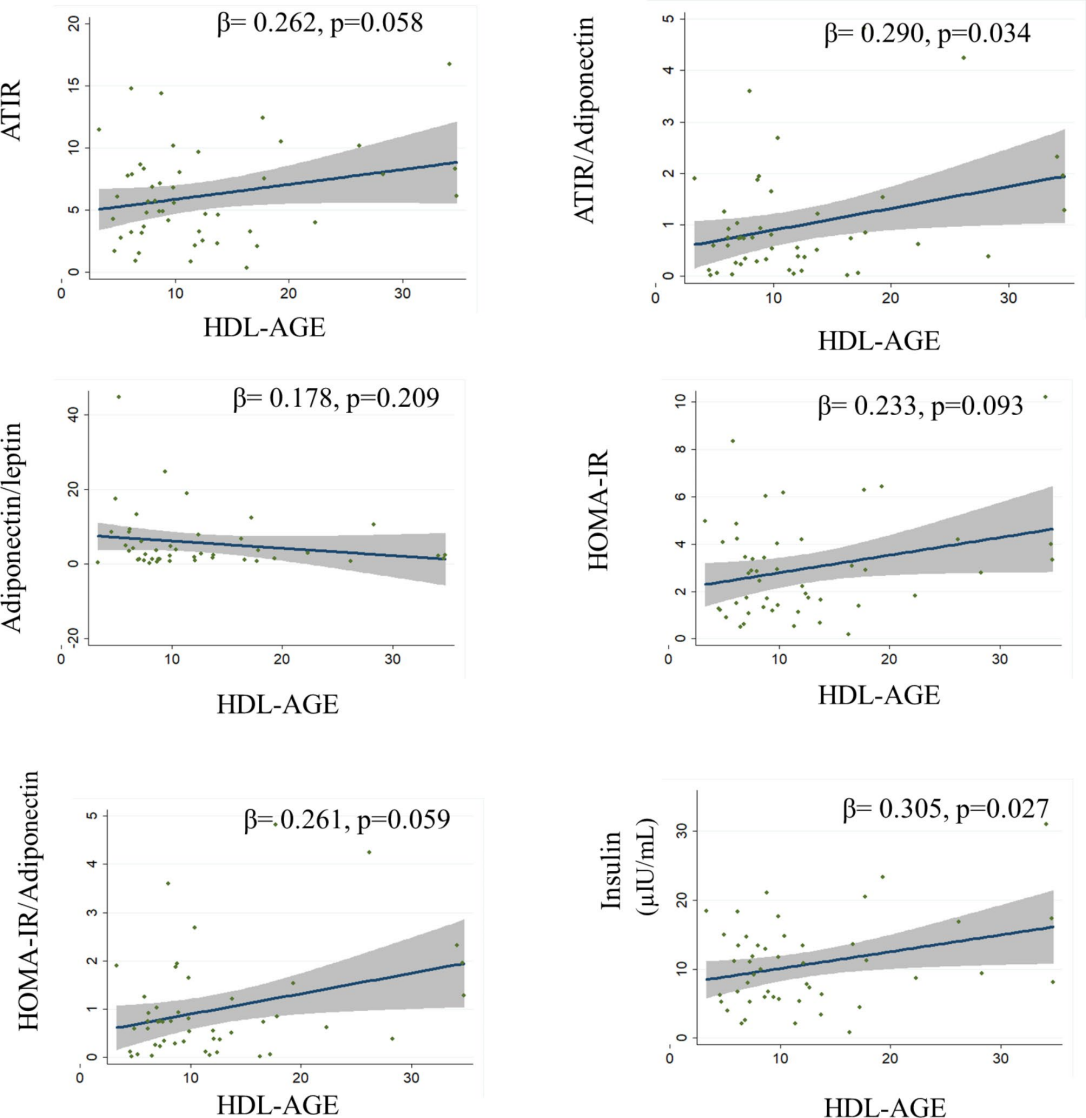
Correlations of HDL-AGE with adipose tissue dysfunction serum markers and inflammation. Analyses were made for the whole population. Values are expressed as the standardized beta coefficient in univariable regression. ATIR: adipose tissue insulin resistance. HOMA-IR; homeostasis model of assessment-insulin resistance; PAI-1; plasminogen activator inhibitor-1

Simple correlations of HDL size and HDL glycation are shown in Figs. 1 and 2, respectively. After adjusting for age, sex, and BMI, HDL size was inversely associated with the ATIR/adiponectin ratio, HOMA/adiponectin ratio, resistin, and PAI-1 and directly associated with HDL-C levels (Table 4, model 1). With further adjustment for HDL-C and triglycerides, the correlations remained significant (Table 4, model 2). In contrast, HDL-AGE was directly correlated with ATIR, the ATIR/adiponectin ratio, insulin, HOMA-IR and HOMA-IR/adiponectin ratio (Table 4, model 1). With further adjustment for HDL-C and triglycerides, the association remained for ATIR, insulin, and HOMA-IR (Table 4, model 2).

**Table 4:** Association of AT dysfunction serum markers, glucose metabolism markers, and inflammation with HDL characteristics

	HDL size (nm)		^a^HDL-AGE (mg-BSA-AGE/mg protein)	
	Model 1	Model 2	Model 1	Model 2
*AT dysfunction markers*				
ATIR	-0.137		0.339*	0.380*
ATIR/adiponectin	-0.387*	-0.281*	0.286*	
Adiponectin/leptin	0.044		-0.212	
Adiponectin (µg/mL)	0.097		-0.238	
*Glucose and lipid metabolism*				
Insulin (µIU/mL)	-0.137		0.448*	0.463*
HOMA-IR	-0.236		0.309*	0.315*
HOMA-IR/Adiponectin	-0.382*	-0.261*	0.294*	
HDL-Cholesterol (mmol/L)	0.466*	0.544*	-0.137	
Triglycerides (mmol/L)	-0.025		0.025	
FFA (mmol/L)	-0.027		0.061	
*Inflammation markers*				
Resistin (ng/mL)	-0.329*	-0.304*	0.096	
PAI-1 (ng/mL)	-0.331*	-0.370*	-0.006	
hs-CRP (mg/L)	-0.077		0.212	

Values are expressed as standardized beta coefficients. *p < 0.005. a) n = 48 for the analysis with HDL-AGE. ATIR: adipose tissue insulin resistance. HOMA-IR; homeostasis model of assessment-insulin resistance; FFA: free fatty acids. PAI-1; plasminogen activator inhibitor-1. hs-CRP: high sensitivity C-reactive protein

Model 1: adjusted for age, sex, and BMI.

Model 2: adjusted for age, sex, BMI, HDL-C, and triglycerides

## Discussion

In the natural history of T2D, the presence of adipose tissue dysfunction can affect HDL metabolism and its chemical structure due to persistent glycation. Our findings show that the ATIR/adiponectin ratio was independently associated with HDL glycation and its subpopulations, whereas ATIR alone was significantly associated with the AGEs concentration in HDLs. On the other hand, inflammatory indicators, such as resistin and PAI-1 were independently associated with HDL size. These data show for the first time that adipose tissue dysfunction, as measured by serum markers, affects the size and glycation of HDL particles in the early T2D status and could have implications for managing and preventing cardiovascular disease.

Mechanisms leading to AT dysfunction are not fully understood because this condition develops alongside comorbidities, such as obesity and insulin resistance. Some AT dysfunction markers could predict the development of HDLs abnormalities in T2D. In the present work, we evaluated three serum markers as indicators of AT dysfunction. Compared to normoglycemic and prediabetic subjects, the ATIR index was significantly higher, whereas the ATIR/adiponectin ratio was marginally higher in subjects with abnormal glucose metabolism. Because the ATIR index considers FFAs and insulin concentrations, it reflects the altered adipose tissue antilipolytic effect of insulin [17]. Thus, the index can identify individuals with AT dysfunction during the early stages of T2D. It has been proposed that ATIR could progressively increase across categories of altered glucose metabolism, from impaired fasting glucose and impaired glucose tolerance to T2D. [19] The above agrees with the results of the present study, as we observed a gradual increase in the ATIR index in subjects with prediabetes and T2D. Although the ATIR/adiponectin ratio reflects inflammation and insulin resistance, it was not associated with T2D, possibly because the difference in adiponectin levels between groups is very subtle (Table 2). Our results align with previous works reporting that plasma adiponectin levels are more related to fat mass excess than the presence of T2D without obesity [30].

Recent interventions have sought to increase the adiponectin/leptin index rather than specifically targeting leptin or adiponectin [8]. Moreover, in patients with overweight and obesity, weight loss is associated with a significant elevation of that index due to a progressive elevation of adiponectin and reduced leptin levels [31]. Recently, in 25 T2D patients who underwent Roux-en-Y gastric bypass, the change in BMI and body fat had the strongest correlation with the adiponectin/leptin ratio. However, this improvement was similar among subjects who had or did not have remission of T2D, indicating that the cardiovascular benefits of the adiponectin/leptin ratio could be due to a reduction in body fat volume or biochemical changes associated with weight reduction [32].

The HDL heterogeneity is a consequence of the maturation process of HDL metabolism, which includes the concerted action of apolipoproteins, transporters, enzymes, and receptors [20]. The present results show that HDL size gradually decreased from normoglycemia to prediabetes and T2D. Furthermore, the ATIR/adiponectin ratio was independently associated with HDL size, indicating that adipose tissue function may influence the metabolism of HDL particles. Previous research has reported evidence of the potential contribution of adipose tissue to HDL metabolism, indicating its ability to maintain cholesterol homeostasis by promoting cholesterol efflux to HDL. In addition, HDL induces the expression of adiponectin through the phosphatidyl inositol kinase-3 (PI3K) pathway in abdominal fat adipocytes [23, 33]. Hence, adipose tissue serum markers may indicate abnormalities in the HDLs subpopulations. The adiponectin levels indicate an increase in low-grade systemic inflammation, which, combined with the ATIR index, could be considered a marker of metaflammation. This type of inflammation has been proposed as the initial mechanism of adipose tissue dysfunction [10]. Nutrient surplus may trigger inflammation by inducing an increase in the number and size of adipocytes. When hypertrophic adipocytes predominate, their size correlates with increased inflammatory adipokine secretion, including leptin, IL-6, IL-8, and MCP-1 [34]. The latter increases the infiltration of macrophages and lymphocytes, exacerbating proinflammatory conditions. This scenario is consistent with the findings of our work, in which we found an association of resistin and PAI-1 with HDL size. Although there are reports that mouse adipocytes produce resistin, a debate about its link to insulin resistance and human obesity is ongoing. However, it has been clearly shown that human adipose tissue-resident macrophages secrete resistin when chronic low-grade inflammation is present, such as in obesity and T2D [35]. Likewise, high concentrations of glucose, insulin, and FFAs induce the expression of PAI-1 [36]. In addition, human adipocytes produce PAI-1 in response to TNF-α derived from adipose tissue macrophages, demonstrating a role for PAI-1 in adipose tissue inflammation [37, 38]. Therefore, metabolic inflammation could contribute directly to HDL size, as serum PAI-1 levels are inversely related to large and intermediate HDL particles in nondiabetic subjects [39].

Compared to our study, a previous report found higher AGE concentrations in subjects with prediabetes [40]. Although there are substantial differences in the sampling method, the values reported in that study were too high, even for subjects with T2D. In this study, patients had a short time since diagnosis (less than four years) and were not receiving pharmacological treatment. In contrast to the observed association with HDL size, glycation of the particles was independently associated with the ATIR and not with the ATIR/adiponectin ratio. Although the atheroprotective role of adiponectin is influenced by lipid metabolism, especially the HDL-C concentration [41], HDL glycation could be due mainly to serum insulin concentrations, which had the strongest association in the multivariable analyses (Table 4).

### Study strengths and limitations

One of the main strengths of this study is the thorough characterization of the subjects, which mitigates potential biases resulting from comorbidities and associated treatments. Moreover, this comprehensive approach facilitated the utilization of non-invasive and practical markers specific to adipose tissue, enabling a robust evaluation of its relationship with HDL metabolism. This study poses limitations represented by the small sample size and the cross-sectional design, which precludes causal inference and may limit statistical power to detect other potential associations. Therefore, the present study should be considered an exploratory analysis, and the findings should be validated in a study involving a larger population. Assessing HDLs and its structural characteristics are time-consuming techniques, and adiponectin and FFAs measurements are not widely available in laboratories, thereby limiting their clinical utility. These results underline the need for standardized current techniques [42] and serum measurements [10, 16, 17] that could be useful as indicators of adipose tissue health status to prevent the development of metabolic abnormalities.

## Conclusion

The HDL size was associated with the ATIR/adiponectin ratio and inflammation, whereas AGEs concentration in HDLs was associated more strongly with the ATIR index. These results suggest that adipose tissue dysfunction could influence HDLs metabolism and integrity. Our findings have important implications for managing and preventing cardiovascular disease in T2D patients.

## Data Availability

The data that support the findings of this study are available from the corresponding author upon reasonable request.

## Abbreviations

AT: Adipose tissue
HDL: High-density lipoproteins
T2D: Type 2 diabetes
AGE: Advanced glycation end-products
FFA: Free fatty acids
PAI-1: Plasminogen activator inhibitor 1
ATIR: Adipose tissue insulin resistance
TNF-alpha: Tumor necrosis factor alfa
IL-6: Interleukin 6
IL-8: Interleukin 8
MCP-1: Monocyte chemoattractant protein 1
HOMA-IR: Homeostatic model assessment of insulin resistance
BMI: Body mass index
hs-CRP: High-sensitive C reactive protein
AST: Aspartate aminotransferase
ALT: Alanine aminotransferase
ApoA1: Apolipoprotein A1
ApoB-100: Apolipoprotein B-100
HbA1C: Hemoglobin A1C
LDL-C: Low-density lipoprotein cholesterol
HDL-C: High-density lipoprotein cholesterol
GFR: Glomerular filtration rate
